# Retracted: Gallic acid from *Terminalia chebula* inhibited the growth of esophageal carcinoma cells by suppressing the Hippo signal pathway

**Published:** 2025

Gui-Li Sun ^1^, Dong Wang ^2^*

^1^ Department of Oncology, Affiliated Hospital of Inner Mongolia University for Nationalities, Tongliao 028000, China

^2^ Department Oncology of Mongolian-Western Medicine, Affiliated Hospital of Inner Mongolia University for Nationalities, Tongliao 028007, China

The Iranian Journal of Basic Medical Sciences states that the article is RETRACTED. This decision is made on the request of the article’s authors because of the concerns raised by PubPeer about the manuscript.



